# Thermal efficiency in hybrid (Al_2_O_3_-CuO/H_2_O) and tri-hybrid (Al_2_O_3_-CuO-Cu/H_2_O) nanofluids between converging/diverging channel with viscous dissipation function: Numerical analysis

**DOI:** 10.3389/fchem.2022.960369

**Published:** 2022-08-25

**Authors:** Adnan, Kamel Guedri, Zehba Raizah, Elsayed Tag-Eldin, Waqas Ashraf, Umar Khan, Ahmed M. Galal

**Affiliations:** ^1^ Department of Mathematics, Mohi-ud-Din Islamic University, Nerian Sharif, AJ&K, Pakistan; ^2^ Mechanical Engineering Department, College of Engineering and Islamic Architecture, Umm Al-Qura University, Makkah, Saudi Arabia; ^3^ Department of Mathematics, College of Science, King Khalid University, Abha, Saudi Arabia; ^4^ Faculty of Engineering and Technology, Future University in Egypt New Cairo, Mansoura, Egypt; ^5^ Departmment of Applied Mathematics and Statistics (AM&S), Institute of Space Technology (IST), Islamabad, Pakistan; ^6^ Department of Mathematics and Statistics, Hazara University Mansehra, Islamabad, Pakistan; ^7^ Mechanical Engineering Department, College of Engineering, Prince Sattam Bin Abdulaziz University, Wadi Addawaser, Saudi Arabia; ^8^ Production Engineering and Mechanical Design Department, Faculty of Engineering, Mansoura University, Mansoura, Egypt

**Keywords:** thermal energy storage, hybrid and modified hybrid nanofluids, thermophysical attributes, engineering applications, mathematical analysis, local energy storage

## Abstract

Heat transfer and energy storage remain a core problem for industrialists and engineers. So, the concept of new heat transfer fluids, namely, nanofluids and hybrid nanofluids, has been introduced so far. Recently, a new third generation of heat transfer fluids has been developed known as modified hybrid nanofluids (MHNs), synthesized by ternary nanomaterials and the host fluid. Therefore, the study was conducted to investigate the energy storage efficiency between (Al_2_O_3_-CuO-Cu/H_2_O)_mhnf_ and (Al_2_O_3_-CuO/H_2_O)_hnf_ in the presence of novel viscous dissipation effects. The problem is developed for a channel with stretchable walls *via* thermophysical attributes of binary and ternary guest nanomaterials and the host liquid. The model is tackled numerically and furnished results for the dynamics, most specifically energy storage efficiency in (Al_2_O_3_-CuO-Cu/H_2_O)_mhnf_. It is examined that the third generation of heat transfer fluids (Al_2_O_3_-CuO-Cu/H_2_O)_mhnf_ has high thermal energy storage efficiency than traditional nano and hybrid nanofluids. Therefore, these new insights in heat transfer would be beneficial and cope with the problems of energy storage in the modern technological world.

## Introduction

The significance of heat transport in the modern technological world is unavoidable due to its remarkable applications. It is a bitter truth that conventional liquids have very limited thermal performance; therefore, these fluids have very limited applications in the modern world era. However, scientists and fluid dynamists thought that how to cope with this core problem. Finally, they introduced the concept of nanofluids. These fluids are the composition of host liquid and guest nanoparticles. The nanoparticles are stably suspended in the liquid and thermally compatible. The majority of issues of the modern world were tackled after the development of nanofluids. However, researchers did not stop their efforts and moved toward the second generation of nanofluids called hybrid nanofluids.

Lately, a superior class of hybrid nanofluids has developed called modified hybrid nanofluid. In this case, further nanoparticles of third guest metals were added to the conventional hybrid nanofluid. The newly suspended additives make the resultant suspension more efficient than hybrid nanofluid. These fluids attained much fame from fluid dynamists and industrialists because of their ultra-high thermal performance than conventional nano and hybrid nanofluids. However, we can categorize the heat transfer fluids into three categories to cope with the heat transfer problems of the modern technological world. These are:

Nanofluids (Choi, 1995) or first-generation heat transfer fluids.

Hybrid nanofluids (Ahmed et al., 2020; Mohyud-Din et al., 2020) or second-generation heat transfer fluids.

Modified hybrid Nanofluids (Abbasi et al., 2021) or third-generation heat transfer fluids.

**FIGURE 1:**
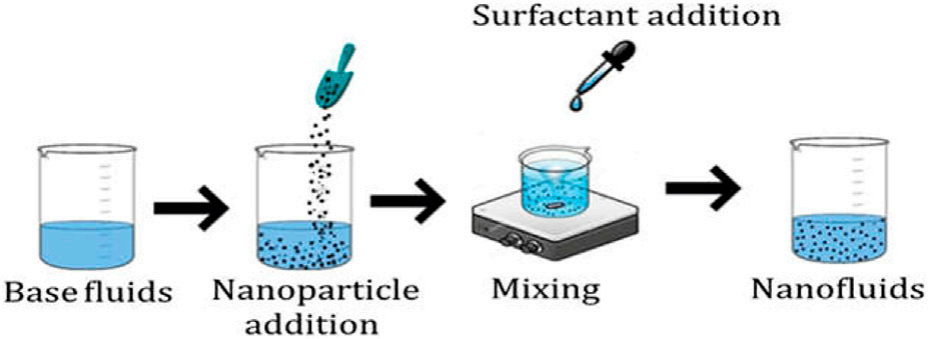
First-generation heat transfer fluids.

**FIGURE 2:**
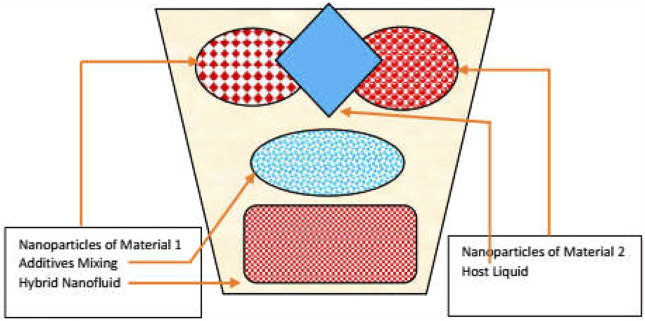
Second-generation heat transfer fluids.

**FIGURE 3:**
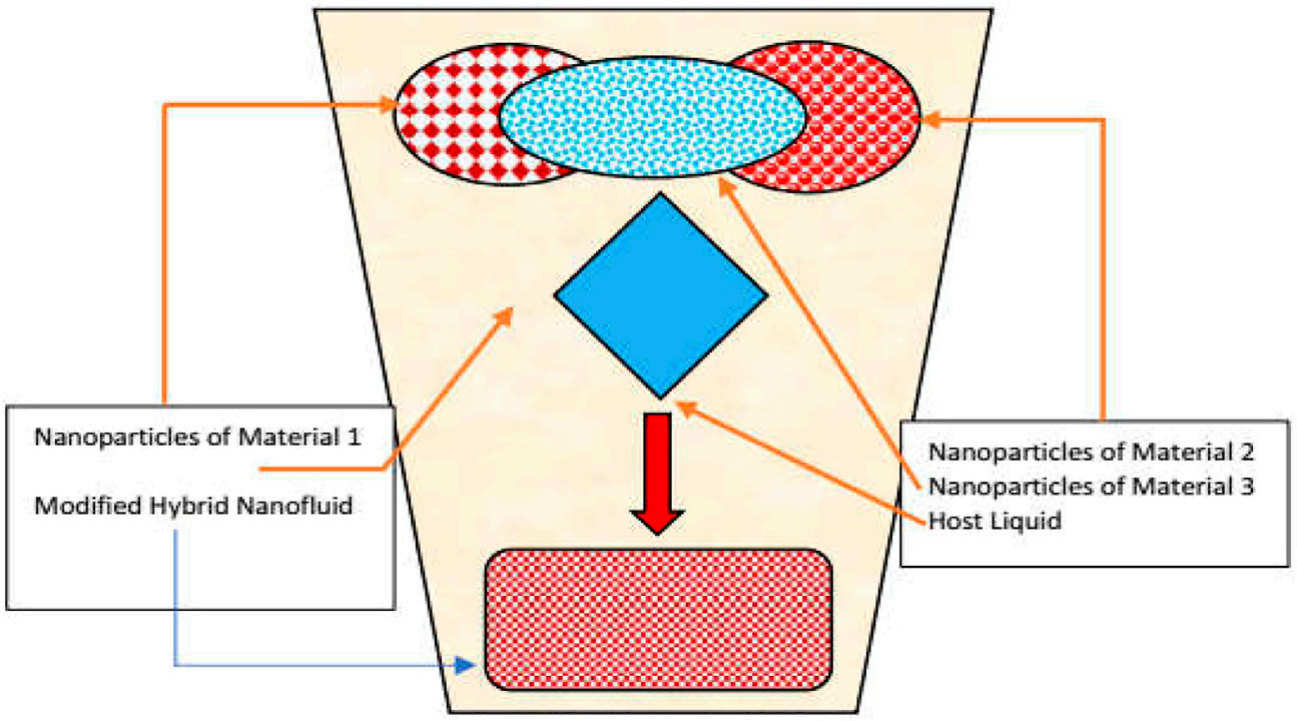
Third-generation heat transfer fluids.

The synthetization process of the aforementioned classes is depicted in Figures 1–3 for nano, hybrid, and modified hybrid nanofluids, respectively.

The newly developed generation (first, second, and third generations) of the fluids strengthen their roots in modern world applications. These could be found in biomedical engineering, electronics, and cooling of the systems to save the drugs and different medicines from moisture in the stores, to check the interaction of biofluids in the human veins and arteries by injecting the hybrid and modified hybrid mixture of nanoparticles, aerodynamics, in the study of chemotherapy, to diagnose cancer symptoms, paint industries, and manufacturing of home appliances. Therefore, the study of heat transfer in nanofluids is significant to accomplish many industrial and engineering processes. In view of such a significant motive, the researchers and fluid dynamists started working in this direction with all of their potential.

The investigation of heat and mass transport mechanisms in opening/narrowing channel is of much interest owing to its applications in medical sciences and engineering as well. Therefore, fluid dynamists focused their attention on exploring the behavior of heat and mass transfer under certain flow assumptions. Such flows extensively appeared in different engineering systems and the flow of blood in human bodies. More specifically, these flows were named Jeffery–Hamel flows after the untiring efforts of Jeffery (1915) and Hamel (1916) during the era of 1915 and 1916, respectively. This concept of flow configuration became very prevalent and conferred the attention of researchers in this direction.

The exploration of thermal performance in the nanofluid under the impacts of internal heat generation/absorption source and viscous dissipation is reported in Akinshilo et al. (2020). The authors organized the study in converging/diverging walls by imposing Lorentz forces on them. The problem is modeled in a cylindrical polar frame, and a dimensionless version is attained *via* feasible similarity transforms. The mathematical section of the work is organized by using the homotopy perturbation method (HPM) and then plotting the results for the concerned flow parameters such as magnetic, Darcy, and Reynolds numbers. It is reported that by increasing the strength of Re, the fluid velocity drops and heat transfer declines at the high Darcy parameter. Although the study is fascinating, researchers performed the results with full consideration; however, it could be prolonged to the next nanofluid generation (hybrid nanofluids) by inducing the influences of Joule heating and thermal radiations.

An analytical study of JH flow for regular liquid is conducted by Patel and Meher (2018). They prolonged the concept of the traditional Adomian decomposition method (ADM) technique to modified Adomian decomposition method (MADM) and solved the problem and found satisfactory results regarding the implementation of the technique. The graphical results were explored and discussed in detail. From the critical review of the article, it is understood that the work has its own significance, but it lacks the important concept of nanofluids and other physical conditions such as slip, thermal jump, and Biot effects. Therefore, more interesting and novel results could be achieved by prolonging the work for hybrid and modified hybrid nanofluids. Further studies on JH flows by taking different physical conditions are reported in Sushila and Shishodia (2014) and Kumbinarasaiah and Raghunatha (2022), and the relevant studies are cited therein.

The applications of nanofluids and hybrid nanofluids attracted researchers and scientists to analyze these fluids for thermal performance, which is a primary element of the modern world. Therefore, numerous studies in this regard have been reported under different flow conditions by using nanofluids synthesized by various base liquids and multiple nano-additives, for instance, the studies by Turkyilmazoglu (2014), Zangooee et al. (2019); Kumar et al. (2021), and Rout et al. (2021). Furthermore, some significant studies related to hybrid nanofluids were reported in Ahmed et al. (2017), Khan et al. (2021) Kumar (2021), Saeed et al. (2021), and Shanmugapriya et al. (2021).

The careful literature survey reveals that comparative heat transfer efficiency of second-generation (Al_2_O_3_-CuO/H_2_O)_hnf_ and third-generation (Al_2_O_3_-CuO-Cu/H_2_O)_mhnf_ nanofluids between opening/narrowing walls subject to the stretching and shrinking conditions has not been reported so far. This type of flow has numerous applications in different engineering systems, most specifically in biomedical engineering. The blood flow at the junction of veins and arteries works under the principle of Jeffery–Hamel (JH) flow. Therefore, the study is organized to explore the velocity, heat transport mechanism, trends in shear stresses, and thermal conductivity in (Al_2_O_3_-CuO/H_2_O)_hnf_ and (Al_2_O_3_-CuO-Cu/H_2_O)_mhnf_ against various parameters, particularly the volumetric fraction. The efficiency of the studied nanofluids can be compared with other reported nanofluids.

## Development of third-generation nanofluid model

### Flow configuration

The flow of (Al_2_O_3_-CuO/H_2_O)_hnf_ and (Al_2_O_3_-CuO-Cu/H_2_O)_mhnf_ subject to viscous dissipation and flexible channel walls is organized between two non-parallel walls. It is supposed that the fluid flow is due to a source/sink positioned at the junction of these two walls. The walls are separated by an angle 2 α is placed in a cylindrical polar frame. The flow is along the only direction with the velocity component $V=\left({\overset{˘}{u}}_{r},0,0\right)$ **.** Furthermore, the velocity at the walls is subject to ${\overset{˘}{u}}_{r}={\overset{˘}{U}}_{w}=\frac{s}{\overset{˘}{r}}$ , where s is the stretching/shrinking rate of the walls. The nanofluids synthesized are subject to the following assumptions:

• The guest hybrid nanomaterials Al_2_O_3_-CuO and H_2_O are thermally compatible.
• The guest hybrid nanomaterial Al_2_O_3_-CuO is uniformly suspended in H_2_O.
• The guest modified hybrid nanomaterials Al_2_O_3_-CuO-Cu and H_2_O are thermally compatible.
• The guest modified hybrid nanomaterial Al_2_O_3_-CuO-Cu is uniformly suspended in H_2_O.

**FIGURE 4:**
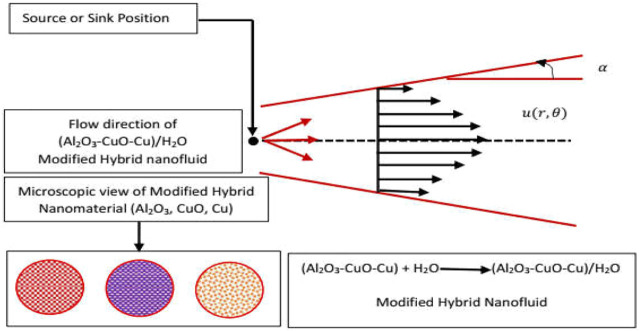
Flow scenario of (Al_2_O_3_-CuO-Cu/H_2_O)_mhnf_.

The physical setup of the flow configuration is depicted in Figure 4.

### Empirical correlations

The nanoparticles of aluminum oxide, copper oxide, and copper are used to synthesize the desired nanofluid (nf), hybrid nanofluid (hnf), and modified hybrid nanofluid (mhnf) in the presence of host liquid water. The empirical correlations for nanofluids, hybrid nanofluids, and modified hybrid nanofluids are given in Tables 1–3, respectively, whereas the shape factor is given in Table 4.

**TABLE 1:** Empirical correlations for first-generation heat transfer fluids.

Characteristics	Empirical correlation
Dynamic viscosity	$\frac{{\overset{˘}{\mu }}_{nf}}{{\overset{˘}{\mu }}_{f}}=\frac{1}{{\left(1-\phi \right)}^{25/10}}$
Effective density	${\overset{˘}{\rho }}_{nf}={\overset{˘}{\rho }}_{f}\left(1-\phi \right)+{\overset{˘}{\rho }}_{s}\phi$
Heat capacity	${\left(\overset{˘}{\rho c_{p}}\right)}_{nf}={\left(\overset{˘}{\rho c_{p}}\right)}_{f}\left(1-\phi \right)+\phi {\left(\overset{˘}{\rho c_{p}}\right)}_{s}$
Thermal conductivity	${\overset{˘}{k}}_{\frac{nf}{{\overset{˘}{k}}_{f}}=\frac{{\overset{˘}{k}}_{s}+\left(\overset{˘}{n}-1\right){\overset{˘}{k}}_{f}-\left(\overset{˘}{n}-1\right)\phi_{s}\left({\overset{˘}{k}}_{f}-{\overset{˘}{k}}_{s}\right)}{{\overset{˘}{k}}_{s}+\left(\overset{˘}{n}-1\right){\overset{˘}{k}}_{f}+\phi_{s}\left({\overset{˘}{k}}_{f}-{\overset{˘}{k}}_{s}\right)}}$
Electrical conductivity	$\frac{{\overset{˘}{\sigma }}_{nf}}{{\overset{˘}{\sigma }}_{f}}=1+\frac{3\left(\frac{{\overset{˘}{\sigma }}_{s}}{{\overset{˘}{\sigma }}_{f}}-1\right)\phi }{\left(\frac{{\overset{˘}{\sigma }}_{s}}{{\overset{˘}{\sigma }}_{f}}+2\;\right)-\left(\frac{{\overset{˘}{\sigma }}_{s}}{{\overset{˘}{\sigma }}_{f}}-1\right)\phi }$
Thermal expansion	${\left(\rho \beta \right)}_{nf}=\left(1-\phi \right){\left(\rho \beta \right)}_{s}+\phi {\left(\rho \beta \right)}_{f}$

**TABLE 2:** Empirical correlations for second-generation heat transfer fluids (hybrid nanofluids) (Ahmed et al., 2021).

Characteristics	Empirical correlation
Dynamic viscosity	$\frac{{\overset{˘}{\mu }}_{\left(Al_{2}O_{3}-CuO\right)water}}{{\overset{˘}{\mu }}_{water}}=\frac{1}{{\left(1-\phi_{1}\right)}^{25/10}{\left(1-\phi_{2}\right)}^{25/10}}$
Effective density	${\overset{˘}{\rho }}_{\left(Al_{2}O_{3}-CuO\right)water}=\left(\left(1-\phi_{2}\right)\left(\left(1-\phi_{1}\right)\rho_{water}+\phi_{1}\rho_{Al_{2}O_{3}}\right)\right)+\phi_{2}\rho_{CuO}$
Heat capacity	${\left(\overset{˘}{\rho c_{p}}\right)}_{\left(Al_{2}O_{3}-CuO\right)water}=\left(1-\phi_{2}\right)\left(\left(1-\phi_{1}\right){\left(\overset{˘}{\rho c_{p}}\right)}_{water}+\phi_{1}{\left(\overset{˘}{\rho c_{p}}\right)}_{Al_{2}O_{3}}\right)+\phi_{2}{\left(\overset{˘}{\rho c_{p}}\right)}_{CuO}$
Thermal conductivity	$\frac{{\overset{˘}{k}}_{\left(Al_{2}O_{3}-CuO\right)water}}{{\overset{˘}{k}}_{nf}}=\frac{{\overset{˘}{k}}_{CuO}+\left(\overset{˘}{n}-1\right){\overset{˘}{k}}_{nf}-\left(\overset{˘}{n}-1\right)\phi_{CuO}\left({\overset{˘}{k}}_{nf}-{\overset{˘}{k}}_{CuO}\right)}{{\overset{˘}{k}}_{CuO}+\left(\overset{˘}{n}-1\right){\overset{˘}{k}}_{nf}+\phi_{CuO}\left({\overset{˘}{k}}_{nf}-{\overset{˘}{k}}_{CuO}\right)},$ where
	$k_{\frac{nf}{k_{f}}=\frac{{\overset{˘}{k}}_{Al_{2}O_{3}}+\left(\overset{˘}{n}-1\right){\overset{˘}{k}}_{water}-\left(\overset{˘}{n}-1\right)\phi_{Al_{2}O_{3}}\left({\overset{˘}{k}}_{water}-{\overset{˘}{k}}_{Al_{2}O_{3}}\right)}{{\overset{˘}{k}}_{Al_{2}O_{3}}+\left(\overset{˘}{n}-1\right){\overset{˘}{k}}_{water}+\phi_{Al_{2}O_{3}}\left({\overset{˘}{k}}_{water}-{\overset{˘}{k}}_{Al_{2}O_{3}}\right)}}$ ;
	$\phi_{Al_{2}O_{3}=\phi_{1};\;\;\phi_{CuO=\phi_{2}}}$
Electrical conductivity	$\frac{{\overset{˘}{\sigma }}_{\left(Al_{2}O_{3}-CuO\right)water}}{{\overset{˘}{\sigma }}_{nf}}=\frac{\sigma_{CuO}+2\sigma_{nf}-2\phi_{CuO}\left(\sigma_{nf}-\sigma_{CuO}\right)}{\sigma_{CuO}+2\sigma_{nf}+\phi_{CuO}\left(\sigma_{nf}-\sigma_{CuO}\right)},$ where
	$\frac{\sigma_{nf}}{\sigma_{water}}=\frac{\sigma_{Al_{2}O_{3}}+2\sigma_{water}-2\phi_{Al_{2}O_{3}}\left(\sigma_{water}-\sigma_{Al_{2}O_{3}}\right)}{\sigma_{Al_{2}O_{3}}+2\sigma_{water}+\phi_{Al_{2}O_{3}}\left(\sigma_{water}-\sigma_{Al_{2}O_{3}}\right)}$
Thermal expansion	${\left(\rho \beta \right)}_{\left(Al_{2}O_{3}-CuO\right)water}=\left(1-\phi_{CuO}\right)\left[\left(1-\phi_{Al_{2}O_{3}}\right){\left(\rho \beta \right)}_{water}+\phi_{Al_{2}O_{3}}{\left(\rho \beta \right)}_{Al_{2}O_{3}}\right]+\phi_{Al_{2}O_{3}}{\left(\rho \beta \right)}_{CuO},$
	where $\phi_{Al_{2}O_{3}}=\phi_{1},\;\;\;\;\phi_{CuO}=\phi_{2}$

**TABLE 3:** Empirical correlations for third-generation heat transfer fluids (modified hybrid nanofluids).

Characteristics	Empirical correlation
Dynamic viscosity	$\frac{{\overset{˘}{\mu }}_{\left(Al_{2}O_{3}-CuO-Cu\right)water}}{{\overset{˘}{\mu }}_{water}}=\frac{1}{{\left(1-\phi_{Al_{2}O_{3}}\right)}^{25/10}{\left(1-\phi_{CuO}\right)}^{25/10}{\left(1-\phi_{Cu}\right)}^{25/10}}$
	$\phi_{Al_{2}O_{3}}=\phi_{1},\;\;\phi_{CuO}=\phi_{2},\;\;\;\phi_{Cu}=\phi_{3}$
Effective density	${\overset{˘}{\rho }}_{\left(Al_{2}O_{3}-CuO-Cu\right)water}=\left(1-\phi_{Cu}\right)\left[\left(1-\phi_{CuO}\right)\left\{\left(1-\phi_{Al_{2}O_{3}}\right)\rho_{water}+\phi_{Al_{2}O_{3}}\rho_{Al_{2}O_{3}}\right\}+\phi_{CuO}\rho_{CuO}\right]+\phi_{Cu}\rho_{Cu},$
	where $\rho_{Al_{2}O_{3}}=\phi_{1},\;\;\phi_{CuO}=\phi_{2},\;\;\;\phi_{Cu}=\phi_{3}\;$
Heat capacity	${\left(\overset{˘}{\rho c_{p}}\right)}_{\left(Al_{2}O_{3}-CuO-Cu\right)water}=\left(1-\phi_{Cu}\right)\left[\left(1-\phi_{CuO}\right)\left\{\left(1-\phi_{Al_{2}O_{3}}\right){\left(\rho c_{p}\right)}_{water}+\phi_{Al_{2}O_{3}}{\left(\rho c_{p}\right)}_{Al_{2}O_{3}}\right\}+\phi_{CuO}{\left(\rho c_{p}\right)}_{CuO}\right]+\rho_{Cu}{\left(\rho c_{p}\right)}_{Cu}$
Thermal conductivity	$\frac{{\overset{˘}{k}}_{\left(Al_{2}O_{3}-CuO-Cuwater\right)}}{{\overset{˘}{k}}_{\left(Al_{2}O_{3}-CuO\right)water}}=\frac{{\overset{˘}{k}}_{Cu}+\left(\overset{˘}{n}-1\right){\overset{˘}{k}}_{\left(Al_{2}O_{3}-CuO\right)water}-\left(\overset{˘}{n}-1\right)\phi_{Cu}\left({\overset{˘}{k}}_{\left(Al_{2}O_{3}-CuO\right)water}-{\overset{˘}{k}}_{Cu}\right)}{{\overset{˘}{k}}_{Cu}+\left(\overset{˘}{n}-1\right){\overset{˘}{k}}_{\left(Al_{2}O_{3}-CuO\right)water}+\phi_{Cu}\left({\overset{˘}{k}}_{\left(Al_{2}O_{3}-CuO\right)water}-{\overset{˘}{k}}_{Cu}\right)}$
	$\frac{k_{\left(Al_{2}O_{3}-CuO\right)water}}{k_{nf}}=\frac{{\overset{˘}{k}}_{CuO}+\left(\overset{˘}{n}-1\right){\overset{˘}{k}}_{nf}-\left(\overset{˘}{n}-1\right)\phi_{CuO}\left({\overset{˘}{k}}_{nf}-{\overset{˘}{k}}_{Cu}\right)}{{\overset{˘}{k}}_{CuO}+\left(\overset{˘}{n}-1\right){\overset{˘}{k}}_{nf}+\phi_{CuO}\left({\overset{˘}{k}}_{nf}-{\overset{˘}{k}}_{Cu}\right)}$
	$\frac{k_{nf}}{k_{f}}=\frac{{\overset{˘}{k}}_{Al_{2}O_{3}}+\left(\overset{˘}{n}-1\right){\overset{˘}{k}}_{water}-\left(\overset{˘}{n}-1\right)\phi_{Al_{2}O_{3}}\left({\overset{˘}{k}}_{water}-{\overset{˘}{k}}_{Al_{2}O_{3}}\right)}{{\overset{˘}{k}}_{Al_{2}O_{3}}+\left(\overset{˘}{n}-1\right){\overset{˘}{k}}_{water}+\phi_{Al_{2}O_{3}}\left({\overset{˘}{k}}_{water}-{\overset{˘}{k}}_{Al_{2}O_{3}}\right)}$
	$\phi_{Al_{2}O_{3}=\phi_{1};\;\;\phi_{CuO=\phi_{2}},\;\;\;\;\phi_{Cu}=\phi_{3},\;\;\;{\overset{˘}{k}}_{\left(Al_{2}O_{3}-CuO\right)water}={\overset{˘}{k}}_{hnf}}$
Electrical conductivity	$\frac{{\overset{˘}{\sigma }}_{\left(Al_{2}O_{3}-CuO-Cu\right)water}}{{\overset{˘}{\sigma }}_{\left(Al_{2}O_{3}-CuO\right)water}}=\frac{{\overset{˘}{\sigma }}_{Cu}+2{\overset{˘}{\sigma }}_{\left(Al_{2}O_{3}-CuO\right)water}-2\phi_{Cu}\left({\overset{˘}{\sigma }}_{\left(Al_{2}O_{3}-CuO\right)water}-{\overset{˘}{\sigma }}_{Cu}\right)}{{\overset{˘}{\sigma }}_{Cu}+2{\overset{˘}{\sigma }}_{\left(Al_{2}O_{3}-CuO\right)water}+\phi_{Cu}\left({\overset{˘}{\sigma }}_{(Al_{2}O_{3}-CuO)water}-{\overset{˘}{\sigma }}_{Cu}\right)}$ , where
	$\frac{{\overset{˘}{\sigma }}_{\left(Al_{2}O_{3}-CuO\right)water}}{{\overset{˘}{\sigma }}_{nf}}=\frac{{\overset{˘}{\sigma }}_{CuO}+2{\overset{˘}{\sigma }}_{nf}-2\phi_{CuO}\left({\overset{˘}{\sigma }}_{nf}-{\overset{˘}{\sigma }}_{CuO}\right)}{{\overset{˘}{\sigma }}_{CuO}+2{\overset{˘}{\sigma }}_{nf}+\phi_{CuO}\left({\overset{˘}{\sigma }}_{nf}-{\overset{˘}{\sigma }}_{CuO}\right)}$
	$\frac{{\overset{˘}{\sigma }}_{nf}}{{\overset{˘}{\sigma }}_{water}}=\frac{{\overset{˘}{\sigma }}_{Al_{2}O_{3}}+2{\overset{˘}{\sigma }}_{water}-2\phi_{Al_{2}O_{3}}\left({\overset{˘}{\sigma }}_{water}-{\overset{˘}{\sigma }}_{Al_{2}O_{3}}\right)}{{\overset{˘}{\sigma }}_{Al_{2}O_{3}}+2{\overset{˘}{\sigma }}_{water}+\phi_{Al_{2}O_{3}}\left({\overset{˘}{\sigma }}_{water}-{\overset{˘}{\sigma }}_{Al_{2}O_{3}}\right)}$
	${\overset{˘}{\sigma }}_{\left(Al_{2}O_{3}-CuO-Cu\right)water}={\overset{˘}{\sigma }}_{mhnf},\;\;{\overset{˘}{\sigma }}_{\left(Al_{2}O_{3}-CuO\right)water}={\overset{˘}{\sigma }}_{hnf}$
	${\overset{˘}{\sigma }}_{Al_{2}O_{3}}={\overset{˘}{\sigma }}_{s1},\;\;{\overset{˘}{\sigma }}_{CuO}={\overset{˘}{\sigma }}_{s2},\;\;{\overset{˘}{\sigma }}_{Cu}={\overset{˘}{\sigma }}_{s3}$

**TABLE 4:** Attributes for different shape factors.

Nanomaterial’s shape	Attribute
Bricks	3.7
Cylinders	4.9
Platelets	5.7
Blades	8.6

The values of guest nanoparticles (Al_2_O_3_, CuO, and Cu) and the host liquid (water) are key ingredients in the study of newly generated heat transfer fluids. These attributes are given in Table 5 for the guest nanoparticles and the host liquid.

**TABLE 5:** Thermophysical values of the guest nanoparticles and the host liquid.

Properties	$\hat{\rho }\left(kg/m^{3}\right)$	${\hat{c}}_{p}\left(J/Kg\;K\right)$	$\hat{k}\left(W/mk\right)$	$\overset{˘}{\sigma }{\left(\text{Ω}m\right)}^{-1}$
Pure water (H_2_O)	997.1	4180	0.6071	$5.5\times 10^{-6}$
Al_2_O_3_	3,970	765	40	$35\times 10^{6}$
Cu	8,933	385	400	$59.6\times 10^{6}$
CuO	6,500	540	18	$6.9\times 10^{-2}$

### Development of modified hybrid nanofluid

The development of the model is based on well-known mass, momentum, and energy constitutive relations in a cylindrical polar frame. For the particular study, these relations are given as follows:

$$\frac{1}{\overset{˘}{r}}\frac{\partial \left(\overset{˘}{r}{\overset{˘}{u}}_{r}\right)}{\partial \overset{˘}{r}}=0,$$ (1)

$${\overset{˘}{\rho }}_{mhnf}\left({\overset{˘}{u}}_{r}\frac{\partial \left({\overset{˘}{u}}_{r}\right)}{\partial \overset{˘}{r}}\right)+\frac{\partial \overset{˘}{p}}{\partial \overset{˘}{r}}-{\overset{˘}{\mu }}_{mhnf}\left(\frac{\partial^{2}{\overset{˘}{u}}_{r}}{\partial {\overset{˘}{r}}^{2}}+\frac{1}{\overset{˘}{r}}\frac{\partial {\overset{˘}{u}}_{r}}{\partial \overset{˘}{r}}+\frac{1}{{\overset{˘}{r}}^{2}}\frac{\partial^{2}{\overset{˘}{u}}_{r}}{\partial {\overset{˘}{\theta }}^{2}}-\frac{{\overset{˘}{u}}_{r}}{{\overset{˘}{r}}^{2}}\right)=0,$$ (2)

$$-\frac{1}{{\overset{˘}{\rho }}_{mhnf}}\frac{\partial \overset{˘}{p}}{\partial \overset{˘}{\theta }}+\frac{2{\overset{˘}{\mu }}_{mhnf}}{{\overset{˘}{r}}^{2}{\overset{˘}{\rho }}_{mhnf}}\frac{\partial {\overset{˘}{u}}_{r}}{\partial \overset{˘}{\theta }}=0,$$ (3)

$${\overset{˘}{u}}_{r}\frac{\partial \overset{˘}{T}}{\partial \overset{˘}{r}}-\frac{{\overset{˘}{k}}_{mhnf}}{{\left(\rho c_{p}\right)}_{mhnf}}\left(\frac{\partial^{2}\overset{˘}{T}}{\partial {\overset{˘}{r}}^{2}}+\frac{1}{\overset{˘}{r}}\frac{\partial \overset{˘}{T}}{\partial \overset{˘}{r}}+\frac{1}{{\overset{˘}{r}}^{2}}\frac{\partial^{2}\overset{˘}{T}}{\partial {\overset{˘}{\theta }}^{2}}\right)-\frac{{\overset{˘}{\mu }}_{mhnf}}{{\left(\rho c_{p}\right)}_{mhnf}}\left(4{\left(\frac{\partial {\overset{˘}{u}}_{r}}{\partial \overset{˘}{r}}\right)}^{2}+\frac{1}{{\overset{˘}{r}}^{2}}{\left(\frac{\partial {\overset{˘}{u}}_{r}}{\partial \overset{˘}{\theta }}\right)}^{2}\right)=0.$$ (4)

The flow conditions that are fixed on the flexible walls are described in the following expressions:

$${\overset{˘}{u}}_{r}=\frac{{\overset{˘}{U}}_{c}}{\overset{˘}{r}}{↓}_{\theta =0},\;\;\frac{\partial {\overset{˘}{u}}_{r}}{\partial \overset{˘}{\theta }}{↓}_{\theta =0}=0,\;\frac{\partial \overset{˘}{T}}{\partial \overset{˘}{\theta }}{↓}_{\theta =0}=0\;,$$ (5)

$${\overset{˘}{u}}_{r}{↓}_{\theta =\pm \alpha }={\overset{˘}{U}}_{w}=\frac{s}{\overset{˘}{r}},\;\;\;\overset{˘}{T}{↓}_{\theta =\pm \alpha }=\frac{{\overset{˘}{T}}_{w}}{{\overset{˘}{r}}^{2}}.$$ (6)

In the constitutive relations, ${\overset{˘}{U}}_{c},\;{\overset{˘}{U}}_{w},\;{\overset{˘}{T}}_{w}$ , and mhnf stands for velocity at the central line, velocity at flexible walls, wall temperature, and modified hybrid nanofluid, respectively. The simplification of mass conservation in a cylindrical polar frame is reduced to the following version:

$$f\left(\overset{˘}{\theta }\right)=\overset{˘}{r}{\overset{˘}{u}}_{r}\;.$$ (7)

Furthermore, for non-dimensionalization of the model, the following similarity relations are introduced:

$$F\left(\eta \right)=\frac{f\left(\overset{˘}{\theta }\right)}{{\overset{˘}{U}}_{c}}\;,\;\;\eta =\frac{\overset{˘}{\theta }}{\alpha },\;\;\;\beta =\frac{\overset{˘}{T}}{{\overset{˘}{T}}_{w}}{\overset{˘}{r}}^{2}.$$ (8)

In the implementation of desired partial differentiation from Eq. 8, in the constitutive model, the following dimensionless model is acquired:

$$F^{\prime \prime \prime }+\frac{2\alpha R_{e}\left[{\left(1-\phi_{Al_{2}O_{3}}\right)}^{\frac{25}{10}}{\left(1-\phi_{CuO}\right)}^{\frac{25}{10}}{\left(1-\phi_{Cu}\right)}^{\frac{25}{10}}\;\right]}{{\left(\left(1-\phi_{Cu}\right)\left[\left(1-\phi_{CuO}\right)\left\{\left(1-\phi_{Al_{2}O_{3}}\right)+\phi_{Al_{2}O_{3}}\rho_{\frac{Al_{2}O_{3}}{\rho_{water}}}\right\}+\phi_{CuO}\rho_{\frac{CuO}{\rho_{water}}}\right]+\frac{\phi_{Cu}\rho_{Cu}}{\rho_{water}}\right)}^{-1}}FF^{\prime }+4\alpha^{2}F^{\prime }=0,$$ (9)

$$\begin{array}{l} \beta^{\prime \prime }+4\alpha^{2}\beta +\frac{\left[\left(1-\phi_{Cu}\right)\left[\left(1-\phi_{CuO}\right)\left\{\left(1-\phi_{Al_{2}O_{3}}\right)+\frac{\phi_{Al_{2}O_{3}}{\left(\rho c_{p}\right)}_{Al_{2}O_{3}}}{{\left(\rho c_{p}\right)}_{water}}\right\}+\frac{\phi_{CuO}{\left(\rho c_{p}\right)}_{CuO}}{{\left(\rho c_{p}\right)}_{water}}\right]+\frac{\rho_{Cu}{\left(\rho c_{p}\right)}_{Cu}}{{\left(\rho c_{p}\right)}_{water}}\right]}{\frac{{\overset{˘}{k}}_{Cu}+\left(\overset{˘}{n}-1\right){\overset{˘}{k}}_{\left(Al_{2}O_{3}-CuO\right)water}-\left(\overset{˘}{n}-1\right)\phi_{Cu}\left({\overset{˘}{k}}_{\left(Al_{2}O_{3}-CuO\right)water}-{\overset{˘}{k}}_{Cu}\right)}{{\overset{˘}{k}}_{Cu}+\left(\overset{˘}{n}-1\right){\overset{˘}{k}}_{\left(Al_{2}O_{3}-CuO\right)water}+\phi_{Cu}\left({\overset{˘}{k}}_{\left(Al_{2}O_{3}-CuO\right)water}-{\overset{˘}{k}}_{Cu}\right)}}\\ \left(2Pr\alpha^{2}F\beta +\frac{PrEc}{R_{e}\left[{\left(1-\phi_{Al_{2}O_{3}}\right)}^{\frac{25}{10}}{\left(1-\phi_{CuO}\right)}^{\frac{25}{10}}{\left(1-\phi_{Cu}\right)}^{\frac{25}{10}}\right]}\left(4\alpha^{2}F^{2}+{F^{\prime }}^{2}\right)\right)=0. \end{array}$$ (10)

The functions F and β in the abovementioned model depend on the variable η . Furthermore, the conditions imposed on the channel walls transformed in the following version after utilizing the similarity equations:

$$F\left(\eta_{=0}\;\right)=1,\;\;\;F^{\prime }\left(\eta_{=0}\right)=0,\;\;\beta^{\prime }\left(\eta_{=0}\right)=0$$

$$F\left(\eta_{=1}\right)=S,\;\;\beta \left(\eta_{=1}\right)=1$$

The parameters involved in the model are summarized in Table 6 with mathematical expressions.

**TABLE 6:** Parameters ingrained in the model with expressions and physical ranges.

Parameter	Name	Expression	Ranges
Reynolds number	Re	$\frac{{\overset{˘}{U}}_{c}\alpha }{{\overset{˘}{\nu }}_{f}}$	Within laminar regimes
Prandtl number	Pr	$\frac{{\left(\rho c_{p}\right)}_{f}{\overset{˘}{U}}_{c}}{{\overset{˘}{k}}_{hnf}}$	6.2
Eckert number	Ec	$Ec=\frac{{\overset{˘}{U}}_{c}^{2}\alpha }{{\overset{˘}{k}}_{hnf}}$	Within physical domain

### Shear stresses and local energy storage

The investigation of shear stresses and local energy storage capability in (Al_2_O_3_-CuO-Cu/H_2_O)_mhnf_ under multiple flow conditions is very imperative from an industrial and engineering point of view. The quantities can be described by the following mathematical formula in the dimensional form:

$$C_{F}=\frac{{\overset{˘}{\mu }}_{\left(Al_{2}O_{3}-CuO-Cu\right)water}\left(\tau_{\overset{˘}{r}\overset{˘}{\theta }}\right)}{{\overset{˘}{\rho }}_{\left(Al_{2}O_{3}-CuO-Cu\right)water}}\;{↓}_{\eta =1},$$ (11)

$$Nu=-\frac{l{\overset{˘}{k}}_{f}\left({\overset{˘}{q}}_{w}\right)}{k{\overset{˘}{T}}_{w}}.$$ (12)

By endorsing the attributes of (Al_2_O_3_-CuO-Cu/H_2_O)_mhnf_ and performing the calculation, the following version is obtained:

$$Re_{r}C_{F}=\frac{{\left[{\left(1-\phi_{Al_{2}O_{3}}\right)}^{\frac{25}{10}}{\left(1-\phi_{CuO}\right)}^{\frac{25}{10}}{\left(1-\phi_{Cu}\right)}^{\frac{25}{10}}\right]}^{-1}F^{\prime }\left(1\right)}{\left[\left(1-\phi_{Cu}\right)\left[\left(1-\phi_{CuO}\right)\left\{\left(1-\phi_{Al_{2}O_{3}}\right)+\frac{\phi_{Al_{2}O_{3}}\left(\rho_{Al_{2}O_{3}}\right)}{\rho_{water}}\right\}+\frac{\phi_{CuO}\left(\rho_{CuO}\right)}{\rho_{water}}\right]+\frac{\phi_{Cu}\left(\rho_{Cu}\right)}{\rho_{water}}\right]},$$ (13)

$$\alpha Nu=-\frac{{\overset{˘}{k}}_{Cu}+\left(\overset{˘}{n}-1\right){\overset{˘}{k}}_{\left(Al_{2}O_{3}-CuO\right)water}-\left(\overset{˘}{n}-1\right)\phi_{Cu}\left({\overset{˘}{k}}_{\left(Al_{2}O_{3}-CuO\right)water}-{\overset{˘}{k}}_{Cu}\right)}{{\overset{˘}{k}}_{Cu}+\left(\overset{˘}{n}-1\right){\overset{˘}{k}}_{\left(Al_{2}O_{3}-CuO\right)water}+\phi_{Cu}\left({\overset{˘}{k}}_{\left(Al_{2}O_{3}-CuO\right)water}-{\overset{˘}{k}}_{Cu}\right)}\beta^{\prime }\left(1\right).$$ (14)

## Mathematical investigation of [(Al_2_O_3_-CuO-Cu)/water]_mhnf_

The mathematical models appearing in the fields of medical sciences, engineering (deflection of beams, load over the bridge, etc.), and biomedical engineering are highly nonlinear in nature. For such models, it is almost impossible to handle the model in the form of an exact solution. However, numerical techniques are best suited to solve and analyze the impacts of various parameters on the dynamics of the model.

The under consideration model is very tedious due to the induction of ternary nanoparticles and their thermophysical attributes; therefore, the numerical technique is helpful to tackle the model and explore the results by altering the flow parameters. For said purpose, we adopted a numerical technique coupled with a shooting algorithm. Primarily, the setup of this technique is based on the development of a first-order initial value problem (IVP) from the higher-order model by means of feasible transformations. After that, the model is then solved by implementing the aforementioned algorithm. The calculation in the under consideration model is very lengthy; therefore, we omit the mathematical procedure. However, the results are plotted against various ranges of the flow parameters and discussed in the next section.

## Results with discussion

### (Al_2_O_3_-CuO-Cu/water)_mhnf_ flow against Re

The Reynolds number, which is a quotient of viscous and inertial forces, is a significant parameter in the study of channel flow. The influences of this parameter on the flow behavior of (Al_2_O_3_-CuO/H_2_O)_hnf_ and (Al_2_O_3_-CuO-Cu/H_2_O)_mhnf_ in stretching/shrinking and opening/narrowing channels are pictured in Figure 2. It is worthy to mention that the values of the opening/narrowing parameter α are taken in degree.

**FIGURE 5:**
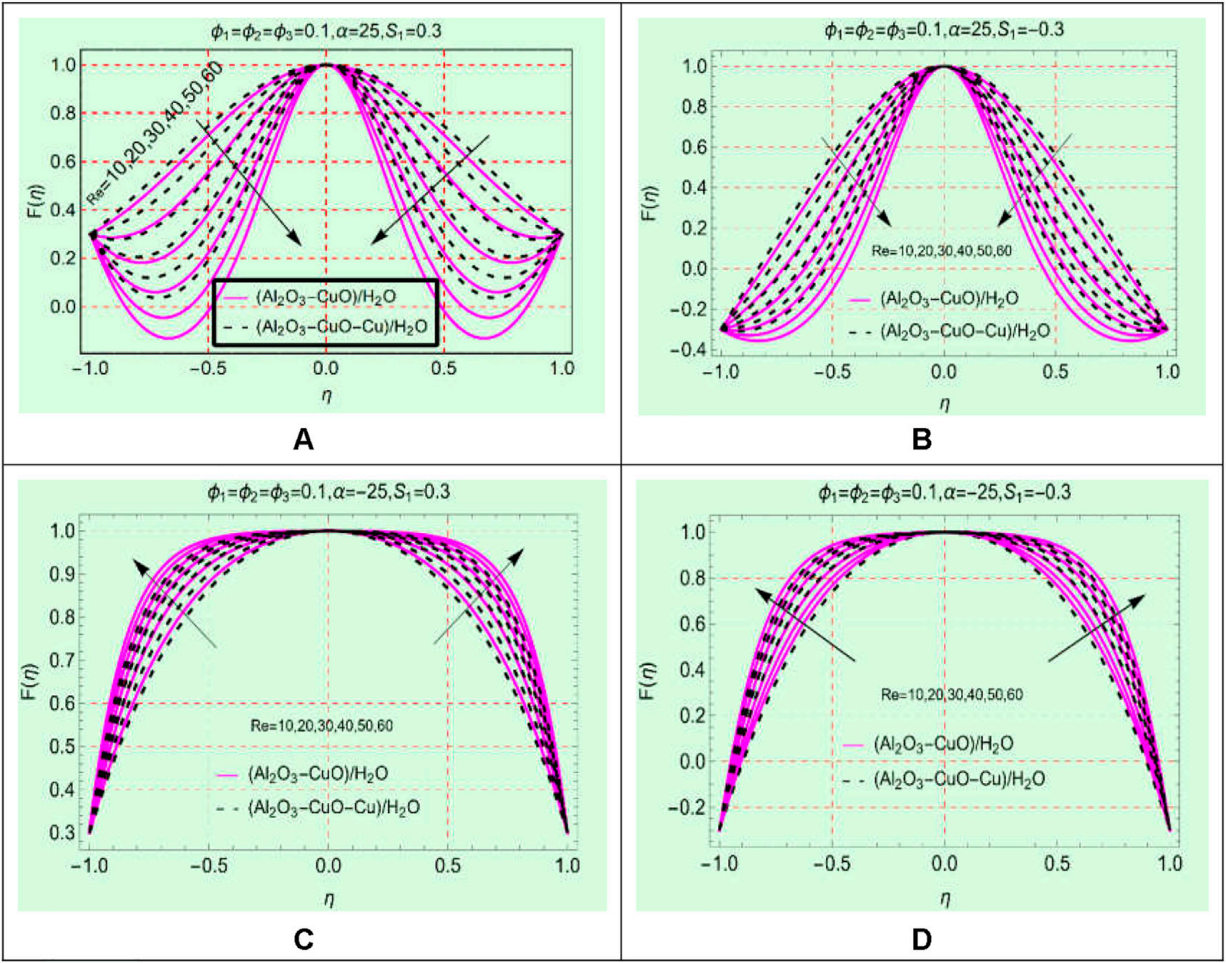
F(η) against Re **(A)** stretching and divergent, **(B)** shrinking and divergent, **(C)** stretching and convergent, and **(D)** shrinking and convergent.

The analysis of Figures 5A and B ensures that the fluid motion drops in a divergent channel for both stretching and shrinking walls. It is noticeable that a backflow phenomenon occurs near the walls because by increasing the Reynolds number, the fluid reverses its motion along the wall instead of mainstream (η=0) . For smaller ranges of Re, the backflow reduces toward the mainstream flow. The maximum fluid motion occurs along the central line for both (Al_2_O_3_-CuO/H_2_O)_hnf_ and (Al_2_O_3_-CuO-Cu/H_2_O)_mhnf_. Furthermore, shrinking of the walls leads to reduced backflow as well.

Figures 5C and D elaborate on the behavior of (Al_2_O_3_-CuO/H_2_O)_hnf_ and (Al_2_O_3_-CuO-Cu/H_2_O)_mhnf_ in the narrowing channel. Physically, the flowing area reduces in the narrowing channel due to which force per unit area enhances which leads to an increment in the motion. The flow profile becomes more flattened at the central position due to the higher strength of Re and narrowing parameter α . The maximum fluid motion is observed near the vicinity of the central portion, and it gradually slows down toward the channel walls.

### (Al_2_O_3_-CuO-Cu/water)_mhnf_ thermal behavior against Ec

**FIGURE 6:**
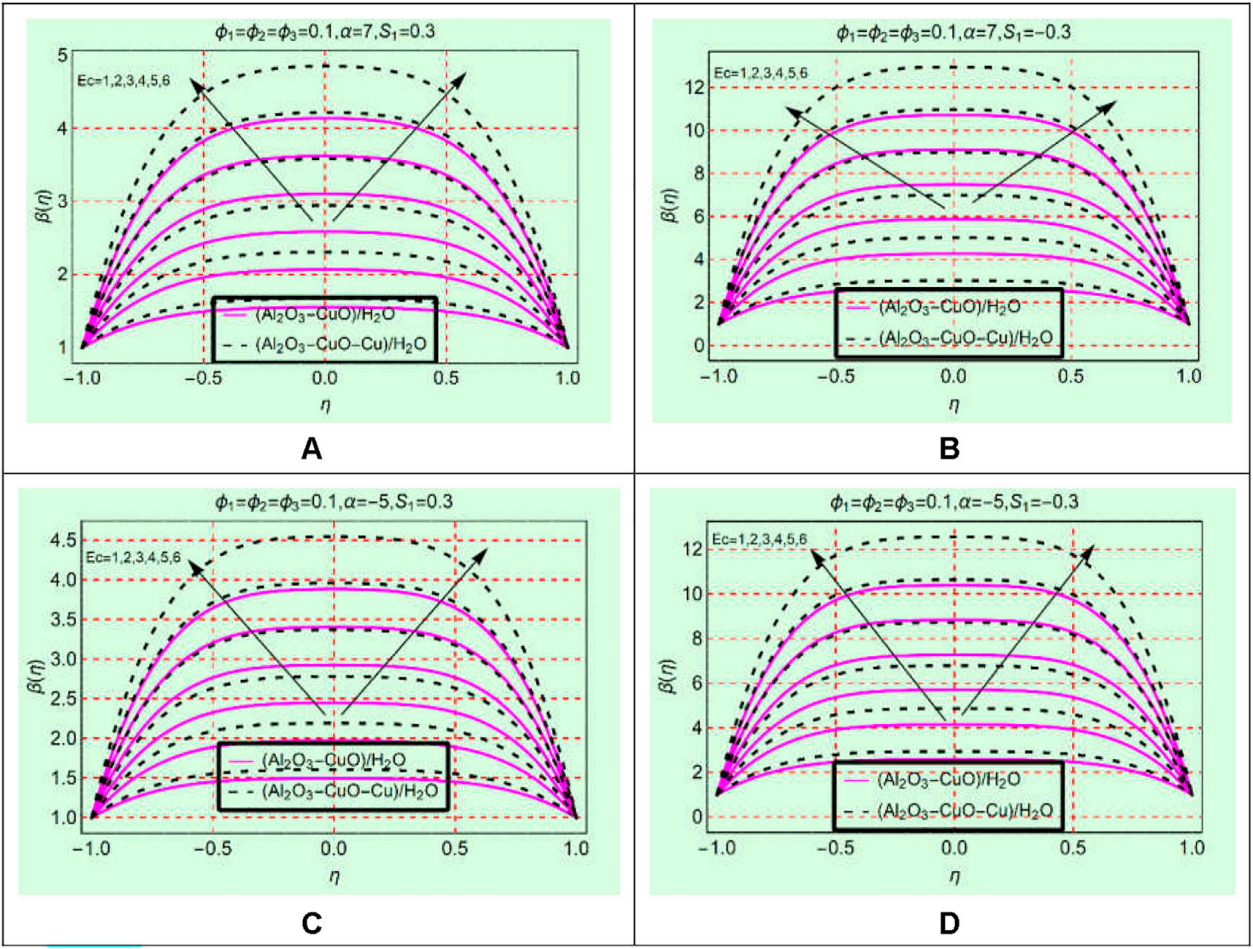
β(η) against Ec **(A)** stretching and divergent, **(B)** shrinking and divergent, **(C)** stretching and convergent, and **(D)** shrinking and convergent.

The viscous dissipation is an important physical phenomenon regarding the energy storage in (Al_2_O_3_-CuO/H_2_O)_hnf_ and (Al_2_O_3_-CuO-Cu/H_2_O)_mhnf_. The Eckert number is a parameter that appeared due to viscous dissipation. Therefore, Figures 3A–D are organized to explore the influences of Ec on the thermal behavior β(η) of (Al_2_O_3_-CuO/H_2_O)_hnf_ and (Al_2_O_3_-CuO-Cu/H_2_O)_mhnf_. From the analysis of Figure 6, it is found that the temperature rises significantly in both sorts of heat transfer fluids for stretching/shrinking and opening/narrowing channels. Physically, the appearance of viscous dissipation enhances the internal energy of the liquids due to which the temperature rises significantly.

The nanofluid containing ternary nanoparticles (Al_2_O_3_-CuO-Cu/H_2_O)_mhnf_ has more capability to store energy than binary-based (Al_2_O_3_-CuO/H_2_O)_hnf_ heat transfer fluids. Physically, the thermal conductivity of (Al_2_O_3_-CuO-Cu/H_2_O)_mhnf_ becomes greater than (Al_2_O_3_-CuO/H_2_O)_hnf_ which increases its energy storage ability. The maximum increasing behavior of binary- and ternary-based nanomaterial liquids is observed along the central line.

### (Al_2_O_3_-CuO-Cu/water)_mhnf_ thermal behavior against Re

**FIGURE 7:**
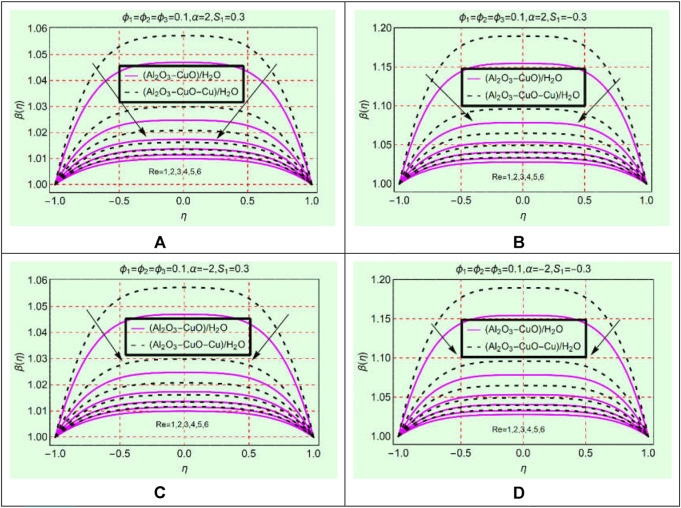
β(η) against Re **(A)** stretching and divergent, **(B)** shrinking and divergent, **(C)** stretching and convergent, and **(D)** shrinking and convergent.

The set of Figures 7A–D elaborates the thermal behavior of (Al_2_O_3_-CuO/H_2_O)_hnf_ and (Al_2_O_3_-CuO-Cu/H_2_O)_mhnf_ against Re. The keen study of Figure 7 reveals that the fluid temperature declines by strengthening Re within the physical domain. The temperature in (Al_2_O_3_-CuO/H_2_O)_hnf_ reduces more abruptly than in (Al_2_O_3_-CuO-Cu/H_2_O)_mhnf_ for both stretching/shrinking and opening/narrowing walls. Physically, (Al_2_O_3_-CuO-Cu/H_2_O)_mhnf_ has high thermal conductivity due to the addition of the third additive Cu due to which its energy storage ability becomes maximum than (Al_2_O_3_-CuO/H_2_O)_hnf_. All these effects are elaborated in Figures 7A–D in both opening and narrowing channels.

### Local energy storage in (Al_2_O_3_-CuO/water)_hnf_ and (Al_2_O_3_-CuO-Cu/water)_mhnf_

**FIGURE 8:**
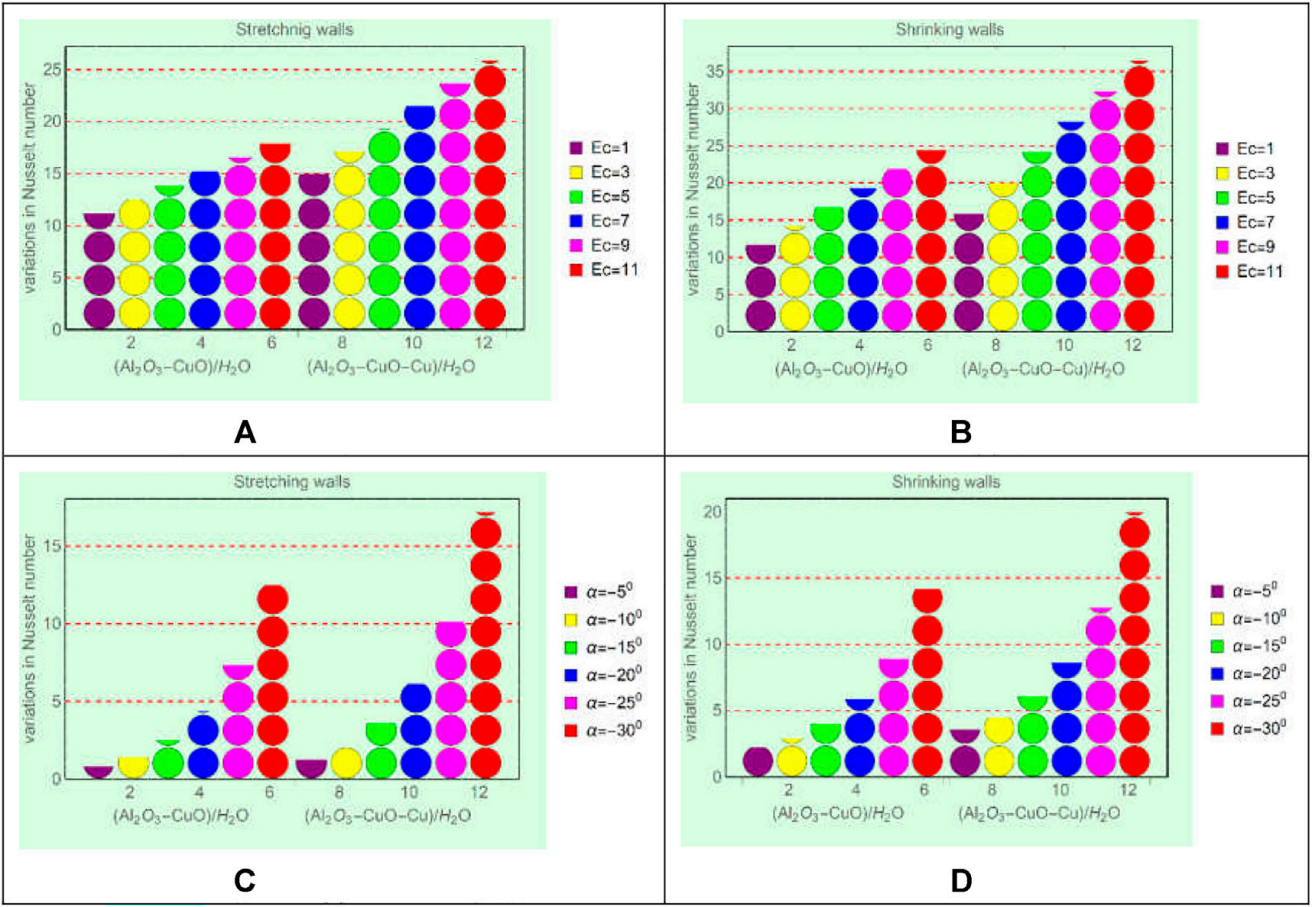
Local energy storage against **(A)** stretching and Ec, **(B)** shrinking and Ec, **(C)** stretching and α , and **(D)** shrinking and α .

**FIGURE 9:**
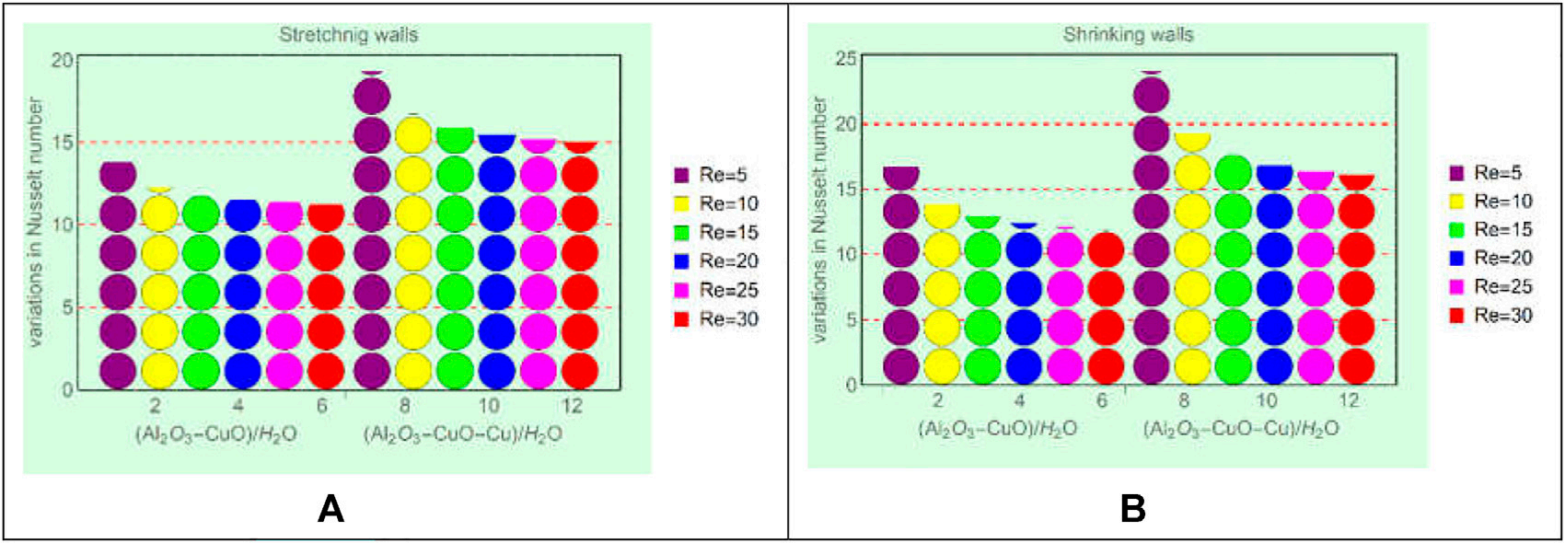
Local energy storage against **(A)** stretching and Re and **(B)** shrinking and Re.

This subsection is devoted to analyzing the local energy storage in (Al_2_O_3_-CuO/H_2_O)_hnf_ and (Al_2_O_3_-CuO-Cu/H_2_O)_mhnf_ for varying flow parameters such as Ec, Re, and α . For said purpose, Figures 8 and 9 displayed over the region of interest.

From Figures 8A–D, it is evident that the local energy storage in (Al_2_O_3_-CuO-Cu/H_2_O)_mhnf_ is higher than that in (Al_2_O_3_-CuO/H_2_O)_hnf_. Physically, the ternary mixture of Al_2_O_3_, CuO, and Cu increases the thermal conductivity of (Al_2_O_3_-CuO-Cu/H_2_O)_mhnf_, while (Al_2_O_3_-CuO/H_2_O)_hnf_ has low thermal conductivity due to the binary mixture of Al_2_O_3_ and CuO. Due to the high thermal conductance of (Al_2_O_3_-CuO-Cu/H_2_O)_mhnf_, the temperature increases rapidly. Moreover, imposed viscous dissipation effects provide extra energy to the fluid, which ultimately boosts the energy ability of (Al_2_O_3_-CuO-Cu/H_2_O)_mhnf_ than (Al_2_O_3_-CuO/H_2_O)_hnf_. Similarly, from Figure 9, it can be seen that drops in the local energy storage in (Al_2_O_3_-CuO-Cu/H_2_O)_mhnf_ is slower than (Al_2_O_3_-CuO/H_2_O)_hnf_. Therefore, modified hybrid nanofluids will be very effective for industrial and engineering applications because of their high energy storage capability.

**FIGURE 10:**
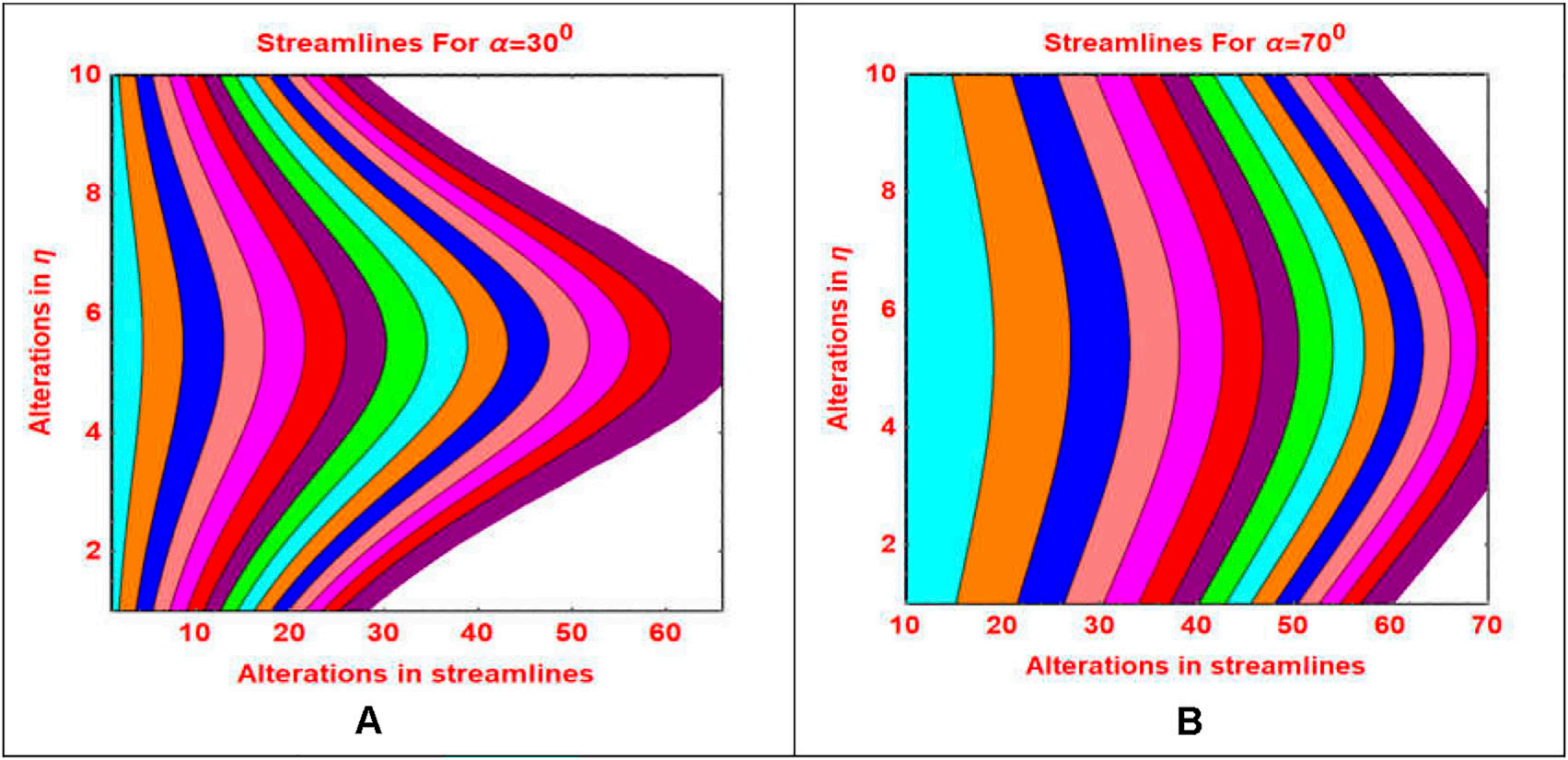
Streamlines pattern for different α values **(A)** α = 30^°^ and **(B)** α = 70^°^.

The streamlines pattern due to α is furnished in Figure 10. It is noted that the streamlines pattern becomes more parabolic shapes for smaller values of α , while it becomes flattened by increasing the value of α .

## Concluding remarks

The study of (Al_2_O_3_-CuO/H_2_O)_hnf_ and (Al_2_O_3_-CuO-Cu/H_2_O)_mhnf_ heat transfer fluids is organized between opening/narrowing channels. The channel walls are allowed to stretch/shrink to some physical extent. The model is developed *via* similarity and NS equations and then solved numerically. The results against the parameters that appeared due to physical phenomena are furnished and discussed deeply in the view of physics behind them. It is found that

• High Reynolds number causes backflow phenomena in the locality of channel walls, and maximum fluid motion is pointed out along the central line.
• The energy storage improved by strengthening viscous dissipation effects and reduces for multiple Re in the model.
• Ternary hybrid nanofluid (Al_2_O_3_-CuO-Cu/H_2_O)_mhnf_ has outstanding heat transport than conventional hybrid due to the addition of the third particle’s volume concentration $\left(\phi_{3}\%\right)$ .
• The optimum thermal behavior in both hybrid and ternary hybrid nanofluids is noticed for shrinking walls, and (Al_2_O_3_-CuO-Cu/H_2_O)_mhnf_ is dominant over (Al_2_O_3_-CuO/H_2_O)_hnf_.
• The local Nusselt number is very high for ternary hybrid nanofluid at various locations inside the channel, and ultra-high thermal conductivity of trihybrid nanoparticles is a key element for this situation.

The presented study revealed that ternary hybrid nanofluid (Al_2_O_3_-CuO-Cu/H_2_O)_mhnf_ has a high temperature featuring an under dissipation function and Reynolds number. Therefore, this class would play a vital role rather than normal hybrid and mono nanofluids in various industries to accomplish the products.

## Data Availability

The raw data supporting the conclusion of this article will be made available by the authors, without undue reservation.
